# Use of recombinant human activated protein C in nonmenstrual staphylococcal toxic shock syndrome

**DOI:** 10.4103/0972-5229.74174

**Published:** 2010

**Authors:** Prashant Nasa, Deepak Sehrawat, Sudha kansal, Rajesh Chawla

**Affiliations:** **From:** Department of Medical ICU, Indraprastha Apollo Hospital, New Delhi, India

**Keywords:** Nonmenstrual toxic shock syndrome, recombinant human activated protein C, staphyloccocal exotoxin, staphylococcal toxic shock syndrome

## Abstract

Toxic shock syndrome (TSS) is a serious, potentially life-threatening condition resulting from an overwhelming immunological response to an exotoxin released by Staphylococcus aureus and group A streptococci. High index of suspicion, early diagnosis and aggressive therapeutic measures must be instituted in view of high mortality of the TSS. In recent years, new agents have been tested to reduce morbidity and mortality in patients with severe sepsis, in addition to standard supportive measures. Among them, recombinant human activated protein C (rhAPC) has been reported to significantly reduce mortality and morbidity in patients with severe sepsis and two or more acute organ failures. We describe our experience with this drug in the early reversal of septic shock from TSS.

## Introduction

Toxic shock syndrome (TSS) is known for its rapid, dramatic and fulminant onset and can lead to significant morbidity and mortality. Quick recognition of the syndrome is important for enabling appropriate and prompt treatment. The authors report a case of severe sepsis in the setting of TSS that responded to treatment with recombinant human activated protein C (rhAPC).

## Case Report

A 27-year-old male, with no significant past history, had painful furuncle in the right axillary region. This was associated with moderate to high-grade fever. He underwent aspiration of the abscess on fourth day of his illness at a private clinic. After the aspiration, he had a sudden and transient (for 1 min) loss of consciousness, which was attributed to a vasovagal attack. This was followed by repeated episodes of vomiting. He was then brought to Indraprastha Apollo hospital, New Delhi, for management.

On admission, he was found to be febrile (temperature 38.4°C), having a diffuse macular erythematous rash, and slight confusion without any focal neurological signs (GCS-14/15). His heart rate was 122/min and BP was 78/30 mm of Hg. He seemed clinically dehydrated and was given fluid challenge of 1 l NS, due to which his BP increased to 98/46 mm of Hg. On local examination, there was a 1-cm diameter, circular, non-tense blister at the right axilla.

He was shifted to ICU, where after placement of a central venous and arterial line, he was further resuscitated as per the surviving sepsis guidelines.[1] He was intubated in view of the septic shock and put on mechanical ventilation. He required noradrenaline 15 μg/min and dopamine 20 μg/kg/min to target his mean arterial pressure of ≥65 mm Hg. The hematological investigation showed hemoglobin 13.7 g% (11.5–16.5 g/dl), total leukocyte count of 19.8 × 10^9^/l (4.0–11.0 × 10^9^/l), with neutrophils 92%, lymphocytes 8% and eosinophils 2%. The plasma biochemical profile revealed serum creatinine of 1.9 mg/dl (0.8–1.1 mg/dl) and urea was 91 mg/dl (15–45 mg/dl). His arterial blood gas (ABG) was showing severe metabolic acidosis with high anion gap of 26 mEq/l, serum lactate 4.2 (<2 mmol/l) and ScvO_2_ 58%. The serum bilirubin was 3.49 mg/dl (0.4–1.1 mg/dl), aspartate transaminase (AST) level was 103 IU/dl (25–40 IU/dl) and alanine transaminase (ALT) level was 190 IU/dl (35–70 IU/dl) and creatine kinase (CK) was 441 U/l, indicating multiorgan dysfunction. His ECHO showed a cardiac index of 3.44 l/min/m^2^, with systemic vascular resistance index (SVRI) of 233.3 dynes/cm^5^/sec/m^2^ with normal left ventricular end-diastolic pressure (LVEDP) and left ventricular ejection fraction (LVEF). His APACHE-II score was 28 and Sequential Organ Failure Assessment (SOFA) score was 10 [Tables 1 and 2]. An abdominal ultrasound revealed no obvious visceral abnormalities. Malaria and dengue were ruled out by microbiological assays.

**Table 1:** Laboratory values on day 2 (before starting rhAPC) and on day 5 (after completing rhAPC)

	Day 2 (before starting rhAPC)	Day 5 (after completing rhAPC)
Hemoglobin (g/dl)	13.7	13
Total leucocyte count (mm^3^)	19,800	11,900
Platelet count (109/l)	1.9	1.28
International Normalised ratio	1.4	1.5
Partial thromboplastin Time test/control (sec)	29.7/33	31/33
Blood urea/serum creatinine (mg/dl)	91/1.9	42/1.2
Sodium(mEq/l)/potassium (mEq/l)/calcium (mg/dl)	143/4.6/10.3	141/3.4/8.7
Uric acid (mg/dl)	6.8	4.9
Serum bilirubin total (mg/dl)	3.49	1.3
Total proteins/albumin (g/dl)	8.2/4.1	7.5/3.7
AST/ALT	103/190	21/25
Alkaline phosphatase (normal 0–75 IU/l)	120	75
Acute physiology and chronic health evaluation II	28	8

**Table 2:** Sequential organ failure assessment scores before and after starting infusion with rhAPC

	Before starting infusion (baseline)	24 hours after infusion	48 hours after infusion	72 hours after infusion	96 hours after infusion
SOFA	10	9	6	6	4

A clinical diagnosis of TSS was made and empirical antibiotic therapy, with specific cover against *Staphylococcus aureus*, was prescribed using vancomycin 1 g i.v. 12 hourly, ceftrioxone 2 g i.v. 12 hourly, and clindamycin 600 mg i.v. 8 hourly after taking two sets of peripheral blood cultures, urine culture and fluid from the blister. He was already on amoxycillin and clavulanic acid, which were started outside the hospital. His condition continued to deteriorate and he had increasing vasopressor requirement and worsening of leukocyte counts. His computed tomography (CT) scan of head was normal, CT of neck showed enlarged bilateral cervical lymph nodes which were not clinically palpable, CT of chest showed minimal bilateral pleural effusion, and CT of abdomen showed mild ascites. Rickettsial serology was performed which turned out to be negative.

On Day 2 of his admission, we decided to use a 96-hour infusion of rhAPC at a rate of 24 μg/kg/hour, in view of worsening multiorgan failure. Fluid from the blister came positive for *Sta. aureus*, which is sensitive to vancomycin and resistant to cloxacillin. Other cultures including blood and urine were negative. His antibiotics were de-escalated and ceftriaxone was stopped. During the next 96 hours, there was improvement in his organ failure with vasopressors weaned off, and APACHE II and SOFA scores decreased to 8 and 4, respectively [Tables 1 and 2]. He was extubated on Day 6 of admission and transferred out of ICU on Day 7.

## Discussion

TSS can be classified on microbiological basis into *Staphylococcus aureus* TSS, *Streptococcus pyogenes* (group A streptococcus) TSS. Staphylococcal TSS was first described by Dr. James K. Todd in 1978.[2] Currently, there are two well-recognized forms of staphylococcal TSS: menstrual and nonmenstrual TSS. Over 90% of menstrual TSS and 60% non-menstrual TSS is associated with a *Sta. aureus* toxic shock syndrome toxin-1 (TSST-1).[3] The mechanism of shock and tissue destruction in TSS is infection by certain strains of *Sta. aureus* and group A streptococci, followed by the production of one or more toxins. These toxins are then absorbed systemically and produce the systemic manifestations of TSS with mediators such as interleukin 1 (IL-1) and tumor necrosis factor (TNF).

The Centre for Disease Control and Prevention has described some diagnostic criteria for Sta. aureus TSS [Table 3].[4,5] They suggested that confirmation of TSS requires all the major criteria listed, plus any three or more of the minor criteria. Our patient fulfilled most of the criteria for TSS case definition proposed by the US Centre for Disease Control and Prevention.[4]

**Table 3:** Criteria for the diagnosis of toxic shock syndrome

Major criteria	Minor criteria (three or more of the following)
Fever >38.8°C	Gastrointestinal (vomiting or diarrhea)
Erythematous rash; skin desquamation, 1–2 weeks after onset of illness	Central nervous system (disorientated or alterations in consciousness without focal neurological signs when fever and hypotension are absent)
Hypotension (systolic<90mmHg)	Mucus membrane hyperemia Muscular (severe myalgia or raised creatine kinase levels at least twice upper limit of normal)
	Hepatic (thrombocytopenia, liver function tests twice upper limit of normal)
	Renal impairment (urea or creatinine twice upper limit of normal)

The management of TSS should be aggressive and includes resuscitation and management bundles of severe sepsis and septic shock.[6] After taking adequate cultures from the wound site and blood, early appropriate antibiotics should be administered. Antibacterial agent selection is complex and subject to change as resistance patterns emerge. Initial empirical antibiotic regimen should include both staphylococcal TSS and streptococcal TSS as differentiating two of them on clinical grounds alone is difficult. Intravenous penicillin G should be administered in addition to a beta-lactamase resistant antibiotic until a bacteriologic diagnosis is confirmed by culture. Alternatively, a first-generation cephalosporin with vancomycin can be used. For patients with group A streptococcal infection, the administration of clindamycin (600–900 mg i.v. q 8 hourly) is recommended because of its multiple effects like inhibiting M protein synthesis, synthesis of penicillin binding proteins, postantibiotic effect and suppression of lipopolysaccharide-induced monocyte synthesis of TNF.[7] Besides using appropriate antibiotics, early source control with debridement of infected/necrotic wounds should also be instituted.

After resuscitation as per Surviving Sepsis Campaign 2008, we could not stabilize the patient with multisystem organ failure, so the decision for rhAPC was then taken in view of septic shock, worsening organ dysfunction and high risk of death (APACHE II > 25).[1] Use of rhAPC as an adjuvant therapy in shock due to TSS has been reported.[8–11] This is the first case report from the Indian subcontinent.

In our report, the vasoactive agents were rapidly weaned following use of rhAPC and the patient had a remarkable recovery with APACHE II score of 8 on its completion. Apart from its evident effect on the coagulation system and the ability of rhAPC to correct the deranged coagulation system in severe sepsis, a series of pleiotropic modulating effects of APC on inflammatory cytokines and cells as well as protective effects on disrupted endothelium have been reported.[12] Since the TSST-1 is a potent inducer of inflammatory markers like IL-1 and TNF which interplay a pivotal role in the pathophysiology of TSS, rhAPC may have had an inhibitory effect on IL-1 levels in patient with TSS.[13] We did not measure IL-1 levels in this patient, and hence, could not observe the effect of rhAPC on IL-1. This response, although short of being complete, may have provided a bridge until antimicrobials could suppress his infection and further diagnostic and therapeutic measures could be initiated.

In conclusion, TSS is a diagnosis of exclusion. Aggressive diagnostic, therapeutic modalities and high clinical suspicion are required in approaching these conditions. The role of Drotrecogin alfa in TSS is still under investigation but could be an option in patients with high risk of death due to septic shock.
